# Supplementary material for: Single cell transcriptomics reveals lineage trajectory of retinal ganglion cells in wild-type and Atoh7-null retinas
Source: Nat Commun. 2021 Mar 5;12:1465. doi: 10.1038/s41467-021-21704-4 (PMC7935890; doi:10.1038/s41467-021-21704-4)
Supplement: Supplementary file 1 — Supplementary Information [file 41467_2021_21704_MOESM1_ESM.pdf]

Suppl. Figure 1

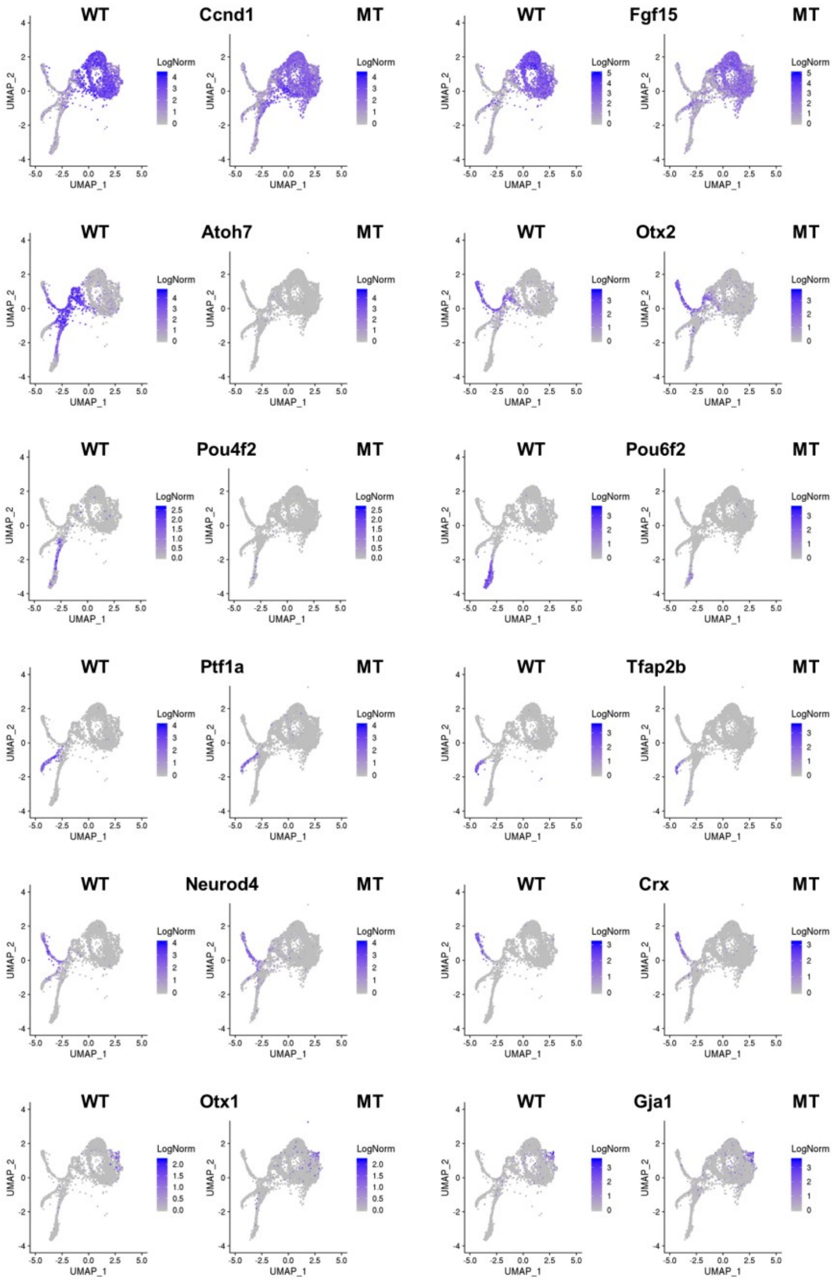

Supplementary Figure 1. Feature plots demonstrating the cluster-specific expression of marker genes in both wild-type (WT) and *Atoh7*-null (MT) retinas at E13.5. These markers were used to assign identities to the clusters, including *Ccnd1* and *Fgf15* for naïve RPCs, *Atoh7* and *Otx2* for transitional RPCs, *Pou4f2* and P*ou6f2* for RGCs, *Ptf1a* and *Tfap2b* for amacrine and horizontal precursors, *Neurod4* and *Crx* for photoreceptors, and *Otx1* and *Gja1* for ciliary margin cells. Note that the expression of *Atoh7* and the two RGC marker genes *Pou4f2* and *Pou6f2* are diminished in the MT cells. Color scales represent natural-log transformed normalized gene expression levels as described in Methods.

Suppl. Figure 2

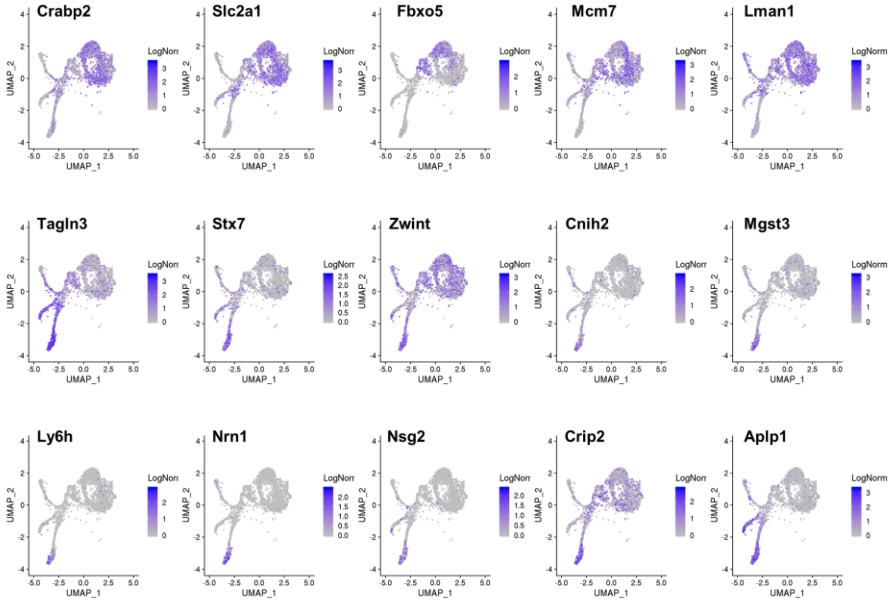

Supplementary Figure 2. Feature plots based on scRNA-seq showing the expression patterns of five naïve RPC enriched genes and ten RGC-enriched genes at E13.5. Comparing with in situ hybridization in the Eurexpress database, scRNA-seq provides more details of cell type-specific expression. For example, *Fbxo5* is expressed only in subsets of naïve RPCs and transitional RPCs, which are likely in the late S and early G2/M phases of the cell cycle. Color scales represent natural-log transformed normalized gene expression levels.

Suppl. Figure 3

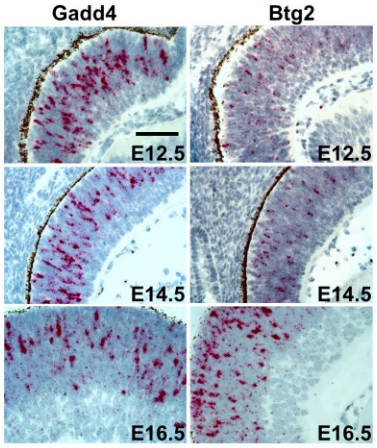

Supplementary Figure 3. In situ hybridization with RNAscope probes confirms that *Gadd45a* and *Btg2* are expressed in subsets of RPCs with patterns similar to *Atoh7* in all three developmental stages (E12.5, E14.5, E16.5) examined. The in situ hybridization experiments were performed three times for each gene. The scale bar is 50 μm.

Suppl. Figure 4

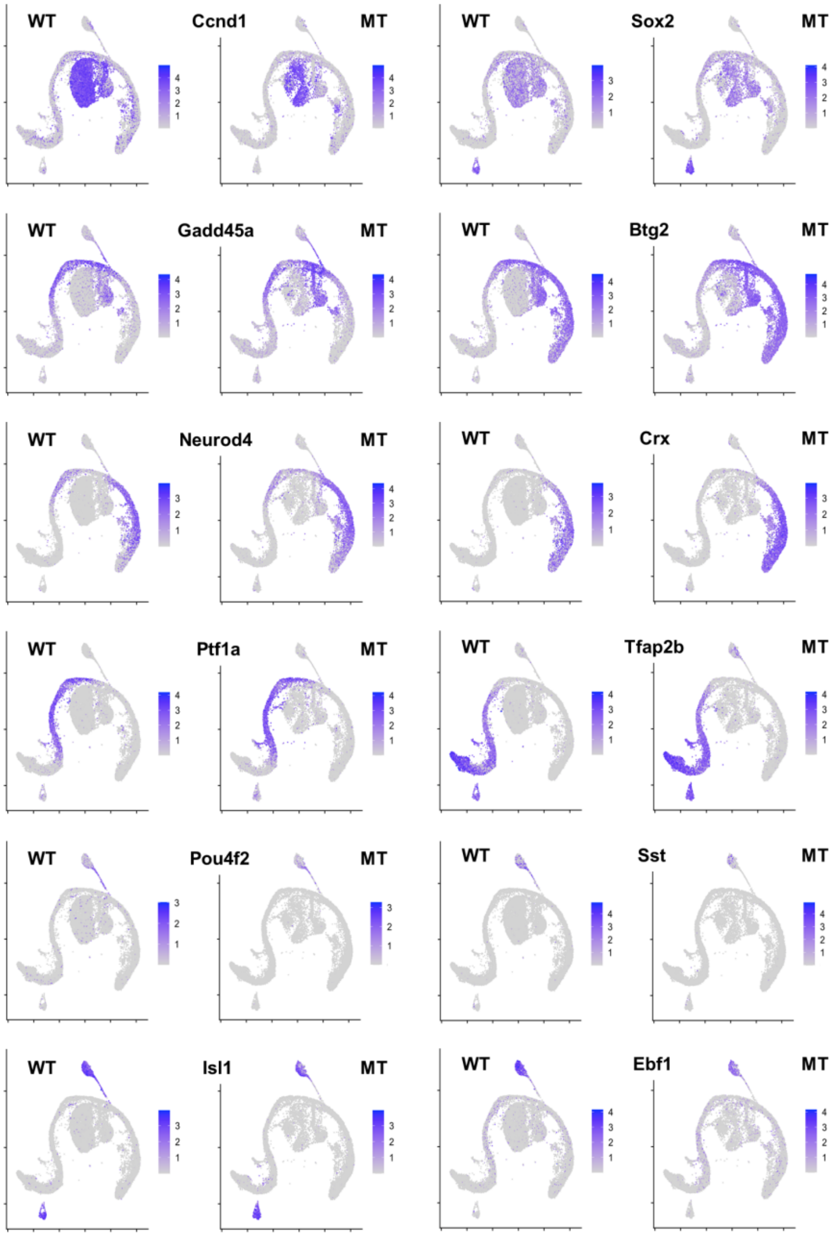

Supplementary Figure 4. Feature plots of additional marker genes to identify clusters of the E17.5 scRNA-seq data. These markers include *Ccnd1* and *Sox2* for naïve RPCs, *Gadd45a* and *Btg2* for transitional RPCs, *Neurod4,* and *Crx* for photoreceptors, *Ptf1a* and *Tfab2b* for horizontal and amacrine cells, and *Pou4f2*, *Sst*, *Isl1*, and *Ebf1* for RGCs. WT is wild-type and MT is *Atoh7*-null. Color scales represent natural-log transformed normalized gene expression levels.

Suppl. Figure 5

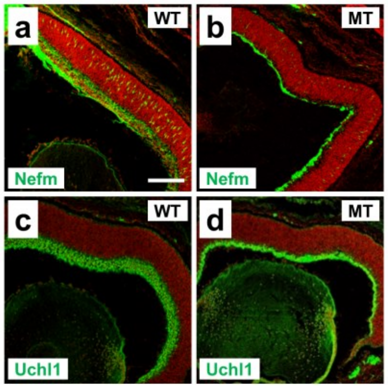

Supplementary Figure 5. Immunofluorescence staining for two RGC markers reveals that some RGCs persist in the *Atoh7*-null retina at E17.5. **a**. **b**. Expression of neurofilament middle chain (Nefm) in wild-type (WT) and *Atoh7*-null (MT) retinas. **c**. **d**. Expression of ubiquitin carboxy-terminal hydrolase L1 (Uchl1) in WT and MT retinas. Red is counterstaining by propidium iodide. For each protein, the immunofluorescence experiment was performed three times with sections from individual animals. The scale bar equals 150 μm.

Suppl. Figure 6

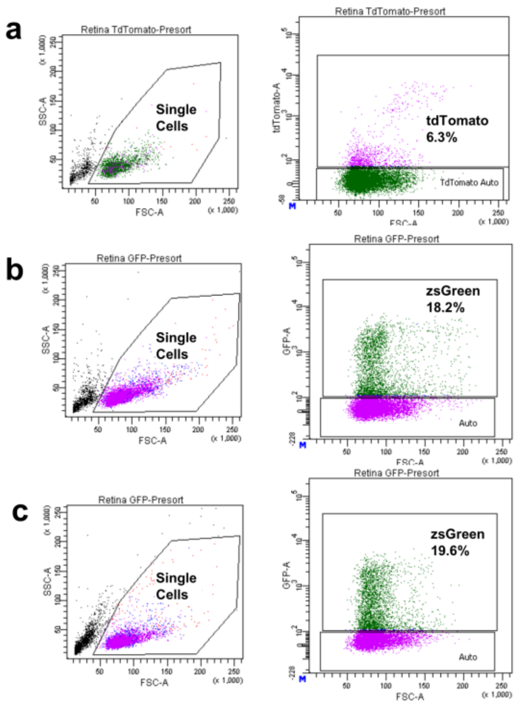

Supplementary Figure 6. Enrichment of E17.5 Atoh7- and Pou4f2-expressing cells by FACS. **a.** Enrichment of *Pou4f2*-expressing cells from E17.5 *Pou4f2*$^{tdTomato/+}$ retinas. **b.** Enrichment of *Atoh7*-expressing cells from E17.5 *Atoh7*$^{zsGreen/+}$ retinas. **c.** Enrichment of *Atoh7*-expressing cells from E17.5 *Atoh7*$^{zsGreen/lacZ}$ retinas. For each genotype, the left is pre-sorting to eliminate dead and aggregated cells, and the right shows the strategy to enrich the desired cell populations base on zsGreen (labeled as GFP) and tdTomato fluorescence. The gating cutoff was set low deliberately to obtain cells in transitional stages and ensure continuity in the scRNA-seq analysis.
