# Supplementary material for: Single cell transcriptomics reveals lineage trajectory of retinal ganglion cells in wild-type and Atoh7-null retinas
Source: Nat Commun. 2021 Mar 5;12:1465. doi: 10.1038/s41467-021-21704-4 (PMC7935890; doi:10.1038/s41467-021-21704-4)
Supplement: Supplementary file 2 — Description of Additional Supplementary Files [file 41467_2021_21704_MOESM2_ESM.pdf]

Description of Additional Supplementary Files

Supplementary Data 1. All genes expressed in E14.5 wild-type retina. Normalized expression levels (counts per million, CPM) for each wild-type replicate (columns WT.1 to WT.3) and their averages are provided. The minimum average CPM is 1 for a gene to be considered expressed.

Supplementary Data 2. Differentially expressed genes (DEGs) in E14.5 *Atoh7*-null, *Pou4f2*-null, and *Isl1*-null retinas. Normalized expression levels (counts per million, CPM) for each replicate of individual genotypes are provided. Log10 fold change (logFC) and FDR for each gene are provided. A gene is considered differentially expressed when it has a minimum fold change of 1.5 and a maximum FDR of 0.05.

Supplementary Data 3. Gene ontology analysis by DAVID of DEGs in E14.5 *Atoh7*-, *Pou4f2*-, and *Isl1*-null retinas. For downregulated DEGs, the combined list from all three genotypes was used. For upregulated genes, DEG lists for individual genotypes were used. In all the analysis, all mouse genes were used as control. The top ten terms based on p values were listed. See Methods for details.

Supplementary Data 4. Genes enriched in individual wild-type clusters. This was achieved using Seurat's FindMarker analysis to report the log fold-change (natural log) of the average expression between cells in individual clusters and the remaining cells. The significance of differences was determined by the Wilcoxon-Rank Sum test to obtain the p-values, and Bonferroni Correction was then performed to correct for multiple testing (p value adjustment, the cutoff is 0.05). PCT.1 and PCT.2 represent the respective percentages of cells in individual clusters and in the remaining cells a gene is detected in.

Supplementary Data 5. Stats of cluster-enriched genes. Counts of enriched gene numbers for all E13.5 clusters are provided. This is derived from Supplementary Data 4.

Supplementary Data 6. Differentially expressed genes (DEGs) in each of the E13.5 *Atoh7*-null clusters as compared to the corresponding wild-type and mutant clusters This was achieved using Seurat's FindMarker analysis to report the log fold-change (natural log) of the average expression between corresponding wild-type and mutant clusters, as described for Supplementary Data 4. The significance of differences was determined by the Wilcoxon-Rank Sum test to obtain the p-values, and Bonferroni Correction was then performed to correct for multiple testing (p value adjustment, the cutoff is 0.05). PCT.1 and PCT.2 represent the respective percentages of cells in the wild-type and mutant clusters each gene is expressed in.

Supplementary Data 7. Gene ontology analysis by DAVID of E13.5 *Atoh7*-null DEGs in naïve RPCs, transitional RPCs, and RGCs. Down- and upregulated genes were analyzed separately for each cluster as described in Methods. The top five relevant GO terms for each list are listed.

Supplementary Data 8. Groups of C5/6 differentially expressed genes (DEGs) and enriched genes by K-means clustering. The grouping of individual genes is based on the K-means results as described in Methods.

Supplementary Data 9. Differentially expressed genes (DEGs) in E17.5 naïve Atoh7-null RPCs, transitional RPCs, and RGCs. This was achieved using Seurat's FindMarker analysis to identify the log fold-change of the average (natural log) expression between corresponding wild-type and mutant clusters, as described for Supplementary Data 4. PCT.1 and PCT.2 represent the respective percentages of cells in the wild-type and mutant clusters that a gene is detected in.
