# Supplementary material for: Single cell transcriptomics reveals lineage trajectory of retinal ganglion cells in wild-type and Atoh7-null retinas
Source: Nat Commun. 2021 Mar 5;12:1465. doi: 10.1038/s41467-021-21704-4 (PMC7935890; doi:10.1038/s41467-021-21704-4)
Supplement: Supplementary file 12 — Reporting summary [file 41467_2021_21704_MOESM12_ESM.pdf]

nature research

Corresponding author(s): Xiuqian Mu

Last updated by author(s): Nov 25, 2020

Reporting Summary

Nature Research wishes to improve the reproducibility of the work that we publish. This form provides structure for consistency and transparency in reporting. For further information on Nature Research policies, see Authors & Referees and the Editorial Policy Checklist .

Statistics

For all statistical analyses, confirm that the following items are present in the figure legend, table legend, main text, or Methods section.

n/a	Confirmed	
☐	☒	The exact sample size (*n*) for each experimental group/condition, given as a discrete number and unit of measurement
☐	☒	A statement on whether measurements were taken from distinct samples or whether the same sample was measured repeatedly
☐	☒	The statistical test(s) used AND whether they are one- or two-sided<br>*Only common tests should be described solely by name; describe more complex techniques in the Methods section.*
☐	☒	A description of all covariates tested
☐	☒	A description of any assumptions or corrections, such as tests of normality and adjustment for multiple comparisons
☐	☒	A full description of the statistical parameters including central tendency (e.g. means) or other basic estimates (e.g. regression coefficient) AND variation (e.g. standard deviation) or associated estimates of uncertainty (e.g. confidence intervals)
☐	☒	For null hypothesis testing, the test statistic (e.g. *F*, *t*, *r*) with confidence intervals, effect sizes, degrees of freedom and *P* value noted<br>*Give P values as exact values whenever suitable.*
☒	☐	For Bayesian analysis, information on the choice of priors and Markov chain Monte Carlo settings
☒	☐	For hierarchical and complex designs, identification of the appropriate level for tests and full reporting of outcomes
☐	☒	Estimates of effect sizes (e.g. Cohen's *d*, Pearson's *r*), indicating how they were calculated

Our web collection on statistics for biologists contains articles on many of the points above.

Software and code

Policy information about availability of computer code

Data collection	Illumina HiSeq2500<br>Demultiplexing: bcl2fastq2
Data analysis	10X Genomics Cellranger 2.1.1<br>Seurat version 3.1.1<br>Scanpy 1.3<br>STAR version 2.6.1d<br>RSEM version 1.3.1<br>EdgeR 3.28.0 in Bioconductor (version 3.10)/R (version 3.6.1)<br>pheatmap (version 1.0.12) in R (version 3.6.1)<br>hclust (included in the pheatmap package)<br>Spot 5.1<br>BD FACSDiva Version 6.1.2

For manuscripts utilizing custom algorithms or software that are central to the research but not yet described in published literature, software must be made available to editors/reviewers. We strongly encourage code deposition in a community repository (e.g. GitHub). See the Nature Research guidelines for submitting code & software for further information.

Data

Policy information about availability of data

All manuscripts must include a data availability statement. This statement should provide the following information, where applicable:

- Accession codes, unique identifiers, or web links for publicly available datasets
- A list of figures that have associated raw data
- A description of any restrictions on data availability

All bulk RNA-seq sequence reads were deposited into the NCBI Short Read Archive with accession numbers SAMN02614558-SAMN02614569 [https://www.ncbi.nlm.nih.gov/bioproject/PRJNA236742]. The scRNA-seq data were deposited into the NCBI Gene Expression Omnibus with an accession number GSE149040 [https://www.ncbi.nlm.nih.gov/geo/query/acc.cgi?acc=GSE149040]. The data are available to the public without restrictions. Source data are provided with this paper.

Field-specific reporting

Please select the one below that is the best fit for your research. If you are not sure, read the appropriate sections before making your selection.

[x] Life sciences [ ] Behavioural & social sciences [ ] Ecological, evolutionary & environmental sciences

For a reference copy of the document with all sections, see nature.com/documents/nr-reporting-summary-flat.pdf

Life sciences study design

All studies must disclose on these points even when the disclosure is negative.

Sample size	The bulk RNA sample sizes followed the guidelines of established by ECODE consortium, and three replicates for each genotype were used. Numbers of cells from each genotype and stage in the scRNA-seq analysis are reported in the text. Whereas there is no consensus regarding the number of cells required for each scRNA-seq experiment, as this is dependent on complexity of cell type composition and sequence depth (Haque A, et al, Genome Med. 2017 Aug 18;9(1):75. doi: 10.1186/s13073-017-0467-4. PMID: 28821273), the number of cells we analyzed for each sample was sufficient to identify all the different cell types/state present in the samples.
Data exclusions	In the bulk RNA-seq analysis, pseudogenes were filtered out to create the final differentiated gene lists, since the functions of most pseudogenes are not known.<br><br>In the scRNA-seq analysis, cells with high unique molecular index counts (nUMI), high mitochondrial transcript load, and high transcript counts for red blood cell markers were filtered out from the analysis. Cells with high nUMI were likely doublets, cells with high mitochondrial transcript load were likely stressed cells caused by tissue processing, and cells with high transcript counts for red blood cell markers were likely contaminated blood cells obtained during tissue collection.<br><br>All these are standard practices in our analysis of RNA-seq and scRNA-seq data from retinal tissues.
Replication	In the bulk RNA-seq, three independent duplicate samples were used for each genotype. All attempts at replication were successful for bulk RNA-seq experiments. For all other experiments described including in situ hybridization and immunofluorescence staining, each was repeated three times independently with success.
Randomization	This is mostly not applicable, as in most cases we only have just two genotypes, wild-type (heterozygous) and mutant mouse retinal samples to compare. However, when multiple replicates were involved, such as in the case of bulk RNA-seq, samples were collected as they became available and pooled randomly according to genotypes into independent biological samples.
Blinding	Sample status was irrelevant to the design as we were testing a wild-type and knockout differences. We confirmed our knockout was present in the appropriate sample before proceeding. Nevertheless, the bioinformaticians performing the analysis were not aware of genotypes or the marker genes used to assign the cluster identifies before finishing the clustering analysis.

Reporting for specific materials, systems and methods

We require information from authors about some types of materials, experimental systems and methods used in many studies. Here, indicate whether each material, system or method listed is relevant to your study. If you are not sure if a list item applies to your research, read the appropriate section before selecting a response.

Materials & experimental systems

n/a	Involved in the study
☐	☒ Antibodies
☒	☐ Eukaryotic cell lines
☒	☐ Palaeontology
☐	☒ Animals and other organisms
☒	☐ Human research participants
☒	☐ Clinical data

Methods

n/a	Involved in the study
☒	☐ ChIP-seq
☐	☒ Flow cytometry
☒	☐ MRI-based neuroimaging

Antibodies

Antibodies used

rabbit anti-Otx2 (1:200, Sigma, HPA000633)
goat anti-Olig2 (1:200, R&D system, AF2418)
goat anti-HA (1:100, Genscript, A00168)
rabbit anti-Atoh7 (1:200, Novus, NBP1-88639)
rabbit anti-Uchl1 (Pgp9.5) (1:500, Millipore, AB1761)
mouse anti-Nefm (1:200, Sigma, N5264)
rabbit anti-Foxn4 21 (Mengqing Xiang Lab)

Validation

rabbit anti-Otx2 (1:200, Sigma, HPA000633, RRID:AB_1079538): This antibody yielded the same immunofluorescence patterns in the retina as previously published in situ and immunohistochemistry patterns (Nishida A, et al, Nat Neurosci. 2003, PMID: 14625556). The manufacturer's validation information can be found at https://www.sigmaaldrich.com/catalog/product/sigma/hpa000633.

goat anti-Olig2 (1:200, R&D system, AF2418, RRID:AB_2157554): This antibody yielded the same immunofluorescence patterns as previously published in situ hybridization data (Hafler BP, et al, Proc Natl Acad Sci U S A. 2012, PMID: 22543161). The manufacturer's validation information can be found at https://resources.rndsystems.com/pdfs/datasheets/af2418.pdf.

goat anti-HA (1:100, Genscript, A00168, RRID:AB_2313847): This antibody was validated by comparing retinas from wild-type and HA-tagged mouse embryos, and by comparing the immunofluorescence patterns with previously published in situ hybridization patterns (Fu X, et al, Dev Dyn. 2009, PMID: 19459208). The manufacturer's validation information can be found at https://www.genscript.com/antibody/A00168-HA_tag_Antibody_pAb_Goat.html.
rabbit anti-Atoh7 (1:200, Novus, NBP1-88639 , RRID:AB_11034390): This antibody was validated previously by comparing the immunofluorescence patterns with an HA-tagged allele, a lacZ knock-in reporter, and previously published in situ hybridization patterns (Miesfeld JB, et al, Gene Expr Patterns. 2018, PMID: 29225067). The manufacturer's validation information can be found at https://www.novusbio.com/PDFs/NBP1-88639.pdf.

rabbit anti-Uchl1 (Pgp9.5) (1:500, Millipore, AB1761, RRID:AB_91019): This antibody was validated by comparing the immunofluorescence patterns with previously published in situ hybridization patterns (Mu X, et al, Dev Biol. 2005, PMID: 15882586). The manufacturer's validation information can be found at https://www.sigmaaldrich.com/catalog/product/mm/ab1761i.

mouse anti-Nefm (1:200, Sigma, N5264, RRID:AB_477278): This antibody was validated by comparing the immunofluorescence patterns with previously published in situ hybridization and immunofluorescence patterns (Mu X, et al, Development. 2004, PMID: 14973295, Mu X, et al, Proc Natl Acad Sci U S A. 2008, PMID: 18460603). The manufacturer's validation information can be found at https://www.sigmaaldrich.com/content/dam/sigma-aldrich/docs/Sigma/Datasheet/4/n5264dat.pdf.

rabbit anti-Foxn4 (Mengqing Xiang Lab): This antibody was previously validated by Dr. Xiang's lab by comparing the immunofluorescence patterns with the in situ hybridization patterns and with that of a lacZ knock-in reporter (Li S, et al Neuron. 2004, PMID: 15363391).

Animals and other organisms

Policy information about studies involving animals; ARRIVE guidelines recommended for reporting animal research

Laboratory animals

The two knockin alleles used in this study, Atoh7zsGreenCREERT2 and Pou4f2FLAGtdTomato, were described in detail in a recent publication (Ge Y, et al, Dev Dyn. 2020, PMID: 32741043). Atoh7zsGreenCREERT2 is a null allele and Pou4f2FLAGtdTomato is a wild-type allele. The other alleles including Atoh7lacZ (null), Pou4f2Gfp (null), the conditional Isl1-null mice (Isl1flox/flox;Six3-Cre), and the Atoh7HA allele were reported before. All these mice were in the C57BL6/129 mixed genetic background. Male and female mice of these strains at two to six months of age were used to obtain embryos of the desired stages through timed mating. Both male and female embryos were used, but were not specifically identified, since sex is not a factor for embryonic retinal development.

	All mice were fed a standard commercial diet and water ad libitum. The animals were maintained at 12 h light/dark cycle and at constant temperature (20 ± 1 °C) and humidity (50 ± 5%).
Wild animals	No wild animals were used in this study.
Field-collected samples	No field collected samples were used.
Ethics oversight	IACUCs of Roswell Comprehensive Cancer Center and University at Buffalo both approved the study.

Note that full information on the approval of the study protocol must also be provided in the manuscript.

Flow Cytometry

Plots

Confirm that:

- [x] The axis labels state the marker and fluorochrome used (e.g. CD4-FITC).
- [x] The axis scales are clearly visible. Include numbers along axes only for bottom left plot of group (a 'group' is an analysis of identical markers).
- [x] All plots are contour plots with outliers or pseudocolor plots.
- [x] A numerical value for number of cells or percentage (with statistics) is provided.

Methodology

Sample preparation	E17.5 mouse retinas with the desired genotypes (Atoh7zsGreenCreERT2/+, Pou4f2tdTomato/+ or Atoh7zsGreenCreERT2/lacZ) were collected in PBS solution, and dissociated by 100 µg/ml trypsin for 10 min in 37 °C, and then quenched with 100 µg/ml soybea trypsin inhibitor. Cells were harvested by centrifuge at 300g for 5min, and resuspended in PBS for cell sorting. No staining was required since the desired cell populations were genetically labeled by fluorescent proteins (zsGreen or tdTomato).
Instrument	BD FACSAria Fusion Cell Sorter
Software	BD FACSDiva Version 6.1.2
Cell population abundance	The tdTomato positive cells are 6.3% of total retina cells in E17.5 embryos of Pou4f2 wild type strain. The zsGreen positive cells were 18.2% and 19.6% of total retina cells in E17.5 Atoh7 wild type and null strains, respectively.
Gating strategy	The first step in gating was distinguishing populations of cells based on their forward and side scatter properties, to exclude debris and aggregated cells. Then single cell gating was performed based on FSC-A x FSC-H properties to eliminate the doublets and other aggregated cells. The zsGreen or tdTomato positive cells were then sorted based on the cell fluorescence intensities. The zsGreen signal was collected by a 488 laser with a 530/30 detector and a 561 laser with a 582/15 detector was used to collect tdTomato signal. The gating thresholds were set just above the background to ensure the continuity of cell populations.

- [x] Tick this box to confirm that a figure exemplifying the gating strategy is provided in the Supplementary Information.
